# Supplementary material for: Association between Exposure to Particulate Matter during Pregnancy and Multidimensional Development in School-Age Children: A Cross-Sectional Study in Italy
Source: Int J Environ Res Public Health. 2021 Nov 5;18(21):11648. doi: 10.3390/ijerph182111648 (PMC8582713; doi:10.3390/ijerph182111648)
Supplement: Supplementary file 1 [file ijerph-18-11648-s001.zip › ijerph-1399617-supplementary.pdf]

Supplementary Material

Figure S1. Geographical distribution of the children included in the study.

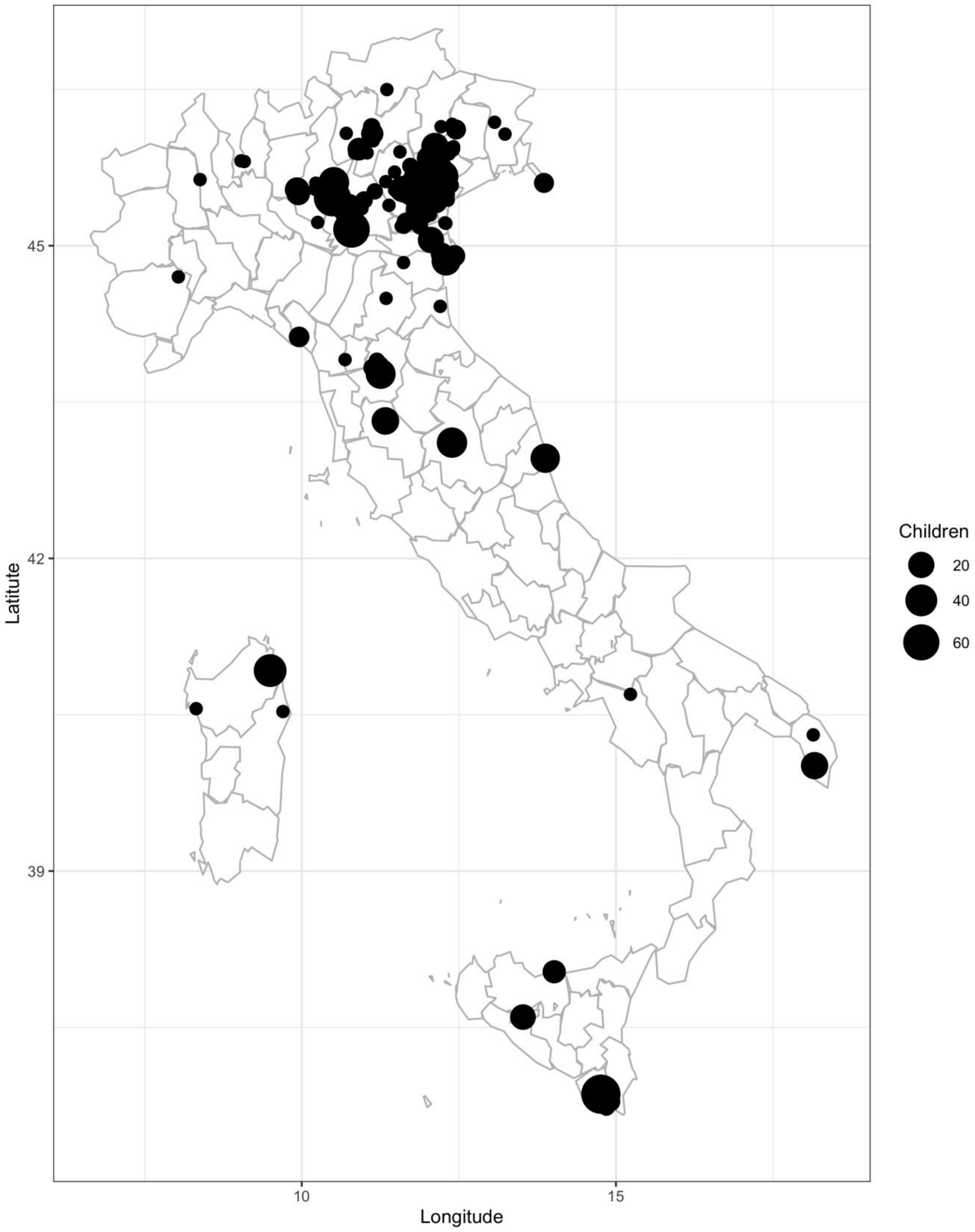

Table S1a. Adjusted* coefficients estimated by ME regression model, relative 95% CI and p-value for Motor, Adaptive and Socio-emotional Score.

	Motor Score			Adaptive Score			Socio-emotional Score		
Predictors	***β***	***95%CI***	***p-value***	***β***	***95%CI***	***p-value***	***β***	***95%CI***	***p-value***
(Intercept)	27.3	25.25 – 29.32	**<0.001**	25.0	22.67 – 27.38	**<0.001**	26.6	24.53 – 28.71	**<0.001**
+1 IQR 1st trim. PM_{10}	0.04	-0.14 – 0.22	0.655	0.19	-0.05 – 0.42	0.117	-0.04	-0.25 – 0.18	0.744
+1 IQR 2nd trim. PM_{10}	-0.03	-0.20 – 0.14	0.728	-0.08	-0.30 – 0.14	0.465	-0.09	-0.28 – 0.10	0.371
+1 IQR 3rd trim. PM_{10}	0.01	-0.16 – 0.19	0.902	0.16	-0.07 – 0.39	0.177	-0.00	-0.21 – 0.21	0.983
Age [1st coef.]	3.09	1.07 – 5.12	**0.003**	1.65	-0.92 – 4.21	0.208	1.65	-0.60 – 3.89	0.151
Age [2nd coef.]	1.61	0.39 – 2.83	**0.010**	1.14	-0.40 – 2.69	0.146	0.84	-0.52 – 2.19	0.225
Age [3rd coef.]	4.61	3.28 – 5.95	**<0.001**	4.27	2.58 – 5.95	**<0.001**	3.10	1.62 – 4.58	**<0.001**
Age [4th coef.]	4.02	2.90 – 5.14	**<0.001**	3.74	2.33 – 5.16	**<0.001**	2.84	1.59 – 4.08	**<0.001**
Age [5th coef.]	5.66	4.16 – 7.15	**<0.001**	6.25	4.35 – 8.15	**<0.001**	4.03	2.36 – 5.69	**<0.001**
Age [6th coef.]	4.16	2.40 – 5.92	**<0.001**	4.92	2.69 – 7.16	**<0.001**	4.00	2.04 – 5.96	**<0.001**
Age [7th coef.]	5.04	2.85 – 7.24	**<0.001**	6.70	3.94 – 9.46	**<0.001**	4.38	1.96 – 6.81	**<0.001**
Gender [Female]	0.21	-0.05 – 0.47	0.116	-0.01	-0.34 – 0.32	0.966	0.12	-0.17 – 0.41	0.416
Job mother [Part-time]	-0.09	-1.82 – 1.65	0.923	-0.06	-2.26 – 2.13	0.955	-0.07	-2.00 – 1.86	0.943
Job mother [Housewife]	0.31	-0.50 – 1.13	0.448	-0.27	-1.30 – 0.76	0.606	-0.61	-1.52 – 0.29	0.183
Job mother [Unemployed/Student]	0.08	-0.65 – 0.80	0.836	-0.17	-1.09 – 0.75	0.718	-0.50	-1.31 – 0.31	0.226
Job father [Part-time/Other]	-0.08	-0.57 – 0.41	0.754	-0.50	-1.14 – 0.13	0.121	-0.22	-0.78 – 0.34	0.449
Ed. level mother [Medium]	0.09	-0.26 – 0.44	0.626	0.12	-0.32 – 0.56	0.597	-0.14	-0.53 – 0.25	0.486
Ed. level mother [High]	0.34	-0.08 – 0.77	0.114	0.67	0.13 – 1.21	**0.015**	0.26	-0.21 – 0.73	0.282
Ed. level father [Medium]	0.20	-0.12 – 0.51	0.222	0.03	-0.37 – 0.43	0.892	0.16	-0.19 – 0.52	0.360
Educ. level father [High]	0.01	-0.43 – 0.44	0.969	-0.16	-0.72 – 0.39	0.558	0.49	0.01 – 0.97	**0.048**
Child nationality [Italian]	0.75	0.01 – 1.50	**0.048**	0.44	-0.50 – 1.39	0.359	0.13	-0.70 – 0.96	0.763
Depr. Index [Very-low]	0.02	-0.56 – 0.60	0.943	-0.84	-1.61 – -0.07	**0.033**	-0.45	-1.14 – 0.24	0.198
Depr. Index [Low]	-0.05	-0.59 – 0.50	0.866	-1.04	-1.76 – -0.32	**0.005**	-0.37	-1.01 – 0.27	0.263
Depr. Index [High]	0.34	-0.39 – 1.06	0.364	-0.64	-1.68 – 0.39	0.221	-0.55	-1.50 – 0.39	0.252
Depr. Index [Very-high]	0.16	-1.25 – 1.57	0.824	0.46	-1.43 – 2.35	0.634	0.45	-1.24 – 2.13	0.604
Siblings [2]	-0.05	-0.41 – 0.32	0.797	0.19	-0.28 – 0.65	0.425	-0.04	-0.45 – 0.37	0.854
Siblings [3+]	0.21	-0.51 – 0.94	0.563	0.69	-0.22 – 1.61	0.139	0.25	-0.55 – 1.06	0.538
Older siblings [Yes]	0.18	-0.11 – 0.46	0.233	-0.23	-0.59 – 0.14	0.221	0.02	-0.30 – 0.34	0.896
Aging Index [124-188]	-0.31	-0.75 – 0.12	0.161	-0.51	-1.12 – 0.10	0.099	-0.49	-1.04 – 0.06	0.080
Aging Index [189-294]	-0.31	-0.77 – 0.14	0.176	-0.71	-1.35 – -0.06	**0.032**	-0.70	-1.28 – -0.11	**0.019**

***Reference category:** *gender [Male], employment status of the mother [Full-time], employment status of the father[Full-time], educational level of the mother [Low], educational level of the father [Low], nationality of the children [non Italian], quintile of deprivation index score [Medium], Siblings [1], Older Siblings [No], tertiles of Aging index [<124].*

Table S1b. Adjusted* coefficients estimated by ME regression model, relative 95% CI and p-value for Cognitive, Communicative and General Score.

	Cognitive Score			Communicative Score			General Score		
Predictors	***β***	***95%CI***	***p-value***	***β***	***95%CI***	***p-value***	***β***	***95%CI***	***p-value***
(Intercept)	27.6	25.61 – 29.51	**<0.001**	23.9	22.04 – 25.67	**<0.001**	130.8	123.6 – 138.0	**<0.001**
+1 IQR 1st trim. PM_{10}	-0.02	-0.22 – 0.17	0.820	0.02	-0.16 – 0.20	0.836	0.27	-0.45 – 0.99	0.468
+1 IQR 2nd trim. PM_{10}	-0.30	-0.48 – -0.12	**0.001**	-0.20	-0.36 – -0.03	**0.018**	-0.72	-1.38 – -0.06	**0.032**
+1 IQR 3rd trim. PM_{10}	-0.31	-0.50 – -0.11	**0.002**	-0.11	-0.29 – 0.07	0.228	-0.27	-0.98 – 0.45	0.464
Age [1st coef.]	2.55	0.47 – 4.64	**0.016**	3.10	1.14 – 5.06	**0.002**	10.91	3.09 – 18.72	**0.006**
Age [2nd coef.]	-0.16	-1.42 – 1.09	0.800	-0.17	-1.35 – 1.01	0.782	3.85	-0.85 – 8.56	0.108
Age [3rd coef.]	5.63	4.26 – 7.01	**<0.001**	5.66	4.37 – 6.95	**<0.001**	22.20	17.06 – 27.34	**<0.001**
Age [4th coef.]	5.74	4.59 – 6.90	**<0.001**	5.04	3.95 – 6.12	**<0.001**	21.43	17.11 – 25.75	**<0.001**
Age [5th coef.]	8.09	6.54 – 9.63	**<0.001**	7.33	5.87 – 8.78	**<0.001**	30.18	24.40 – 35.97	**<0.001**
Age [6th coef.]	10.04	8.22 – 11.86	**<0.001**	7.06	5.35 – 8.77	**<0.001**	30.34	23.53 – 37.16	**<0.001**
Age [7th coef.]	10.49	8.24 – 12.73	**<0.001**	8.21	6.10 – 10.32	**<0.001**	33.68	25.26 – 42.10	**<0.001**
Gender [Female]	-0.18	-0.45 – 0.09	0.197	0.16	-0.10 – 0.41	0.224	0.44	-0.56 – 1.45	0.388
Job mother [Part-time]	-0.32	-2.11 – 1.46	0.722	0.34	-1.34 – 2.02	0.688	-0.31	-7.01 – 6.40	0.929
Job mother [Housewife]	-0.47	-1.30 – 0.37	0.277	-0.61	-1.40 – 0.18	0.129	-1.73	-4.88 – 1.41	0.280
Job mother [Unemployed/Student]	-0.47	-1.22 – 0.28	0.221	-0.37	-1.07 – 0.34	0.309	-1.56	-4.37 – 1.24	0.276
Job father [Part-time/Other]	-0.97	-1.49 – -0.45	**<0.001**	-0.68	-1.17 – -0.19	**0.006**	-2.42	-4.36 – -0.48	**0.014**
Ed. level mother [Medium]	-0.27	-0.64 – 0.09	0.135	0.04	-0.30 – 0.37	0.838	0.02	-1.33 – 1.38	0.974
Ed. level mother [High]	0.11	-0.33 – 0.55	0.620	0.31	-0.11 – 0.72	0.146	2.06	0.41 – 3.70	**0.014**
Ed. level father [Medium]	0.15	-0.18 – 0.47	0.382	0.06	-0.25 – 0.37	0.705	0.54	-0.68 – 1.77	0.383
Ed. level father [High]	0.40	-0.04 – 0.85	0.077	0.47	0.05 – 0.90	**0.027**	1.13	-0.55 – 2.81	0.187
Child nationality [Italian]	0.59	-0.18 – 1.36	0.135	0.66	-0.06 – 1.38	0.074	2.32	-0.56 – 5.20	0.114
Depr. Index [Very-low]	-0.55	-1.20 – 0.09	0.092	-0.04	-0.64 – 0.56	0.887	-1.75	-4.12 – 0.61	0.146
Depr. Index [Low]	-0.20	-0.80 – 0.40	0.517	-0.03	-0.59 – 0.53	0.920	-1.72	-3.93 – 0.48	0.125
Depr. Index [High]	0.27	-0.62 – 1.16	0.553	0.78	-0.04 – 1.59	0.061	-0.15	-3.33 – 3.02	0.925
Depr. Index [Very-high]	1.03	-0.55 – 2.61	0.201	0.40	-1.06 – 1.87	0.588	2.72	-3.06 – 8.50	0.356
Sibilings [2]	0.10	-0.28 – 0.48	0.612	-0.01	-0.37 – 0.34	0.948	0.14	-1.27 – 1.56	0.843
Sibilings [3+]	-0.09	-0.84 – 0.65	0.805	0.69	-0.01 – 1.39	0.054	1.71	-1.08 – 4.50	0.231
Older siblings [Yes]	-0.10	-0.40 – 0.20	0.498	-0.17	-0.45 – 0.11	0.237	-0.32	-1.44 – 0.79	0.569
Aging Index [124-188]	-0.36	-0.87 – 0.16	0.177	-0.60	-1.08 – -0.13	**0.013**	-2.44	-4.30 – -0.58	**0.010**
Aging Index [189-294]	-0.28	-0.84 – 0.27	0.312	-0.58	-1.08 – -0.07	**0.026**	-2.18	-4.16 – -0.19	**0.032**

***Reference category:** *gender [Male], employment status of the mother [Full-time], employment status of the father[Full-time], educational level of the mother [Low], educational level of the father [Low], nationality of the children [non Italian], quintile of deprivation index score [Medium], Siblings [1], Older Siblings [No], tertiles of Aging index [<124].*
